# Supplementary material for: Integrative functional genomics analysis identifies pleiotropic genes for vascular diseases
Source: Nat Commun. 2026 Feb 5;17:3376. doi: 10.1038/s41467-026-69273-8 (PMC13066215; doi:10.1038/s41467-026-69273-8)
Supplement: Supplementary file 1 — Supplementary Information [file 41467_2026_69273_MOESM1_ESM.pdf]

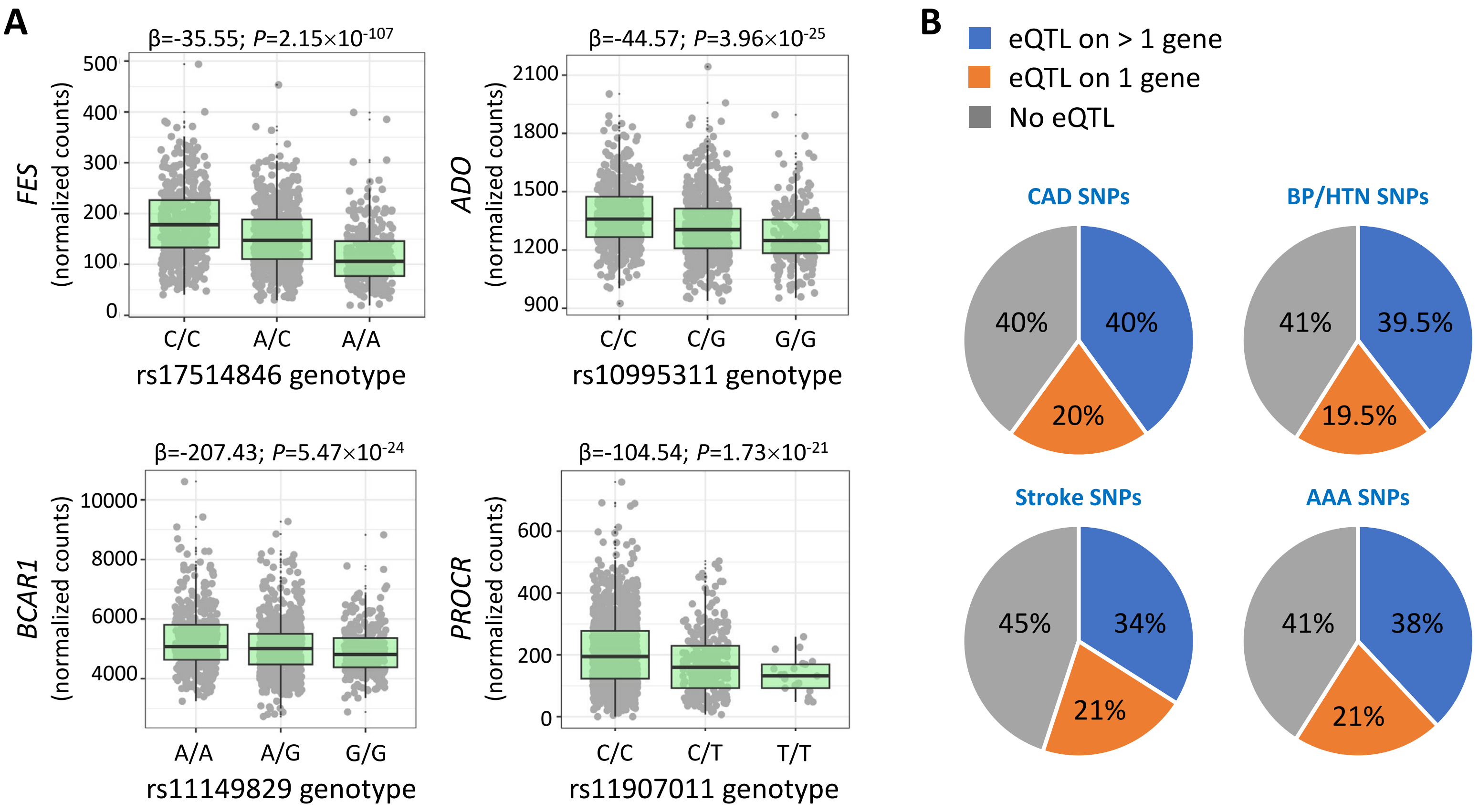

Supplementary Figure 1. eQTL effects in VSMCs from SNPs associated with CAD, blood pressure/hypertension, AAA, or stroke

A. Scatter plots of gene expression accounts by genotype of selected SNPs and genes as examples. **B**. Percentages of SNPs associated with CAD, blood pressure/hypertension (BP/HTN), abdominal aortic aneurysm (AAA), or stroke, that had eQTL effect on >1 gene, eQTL effect on just 1 gene, or no eQTL effect, in VSMCs.

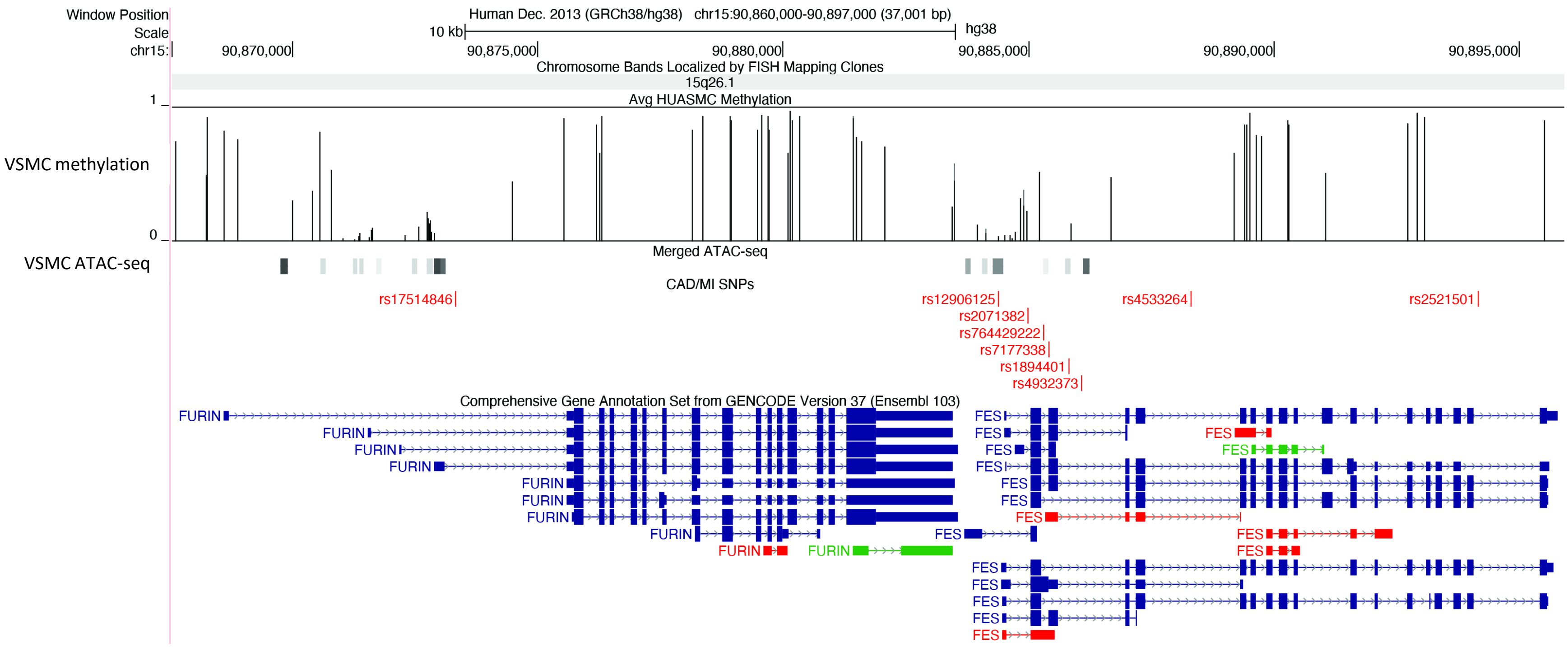

Supplementary Figure 2. Representative results from ATAC-seq and DNA methylation analysis of vascular smooth muscle cells

Shown in the figure are data in the genomic region containing the genetic variant rs1894401 and the *FES* gene.

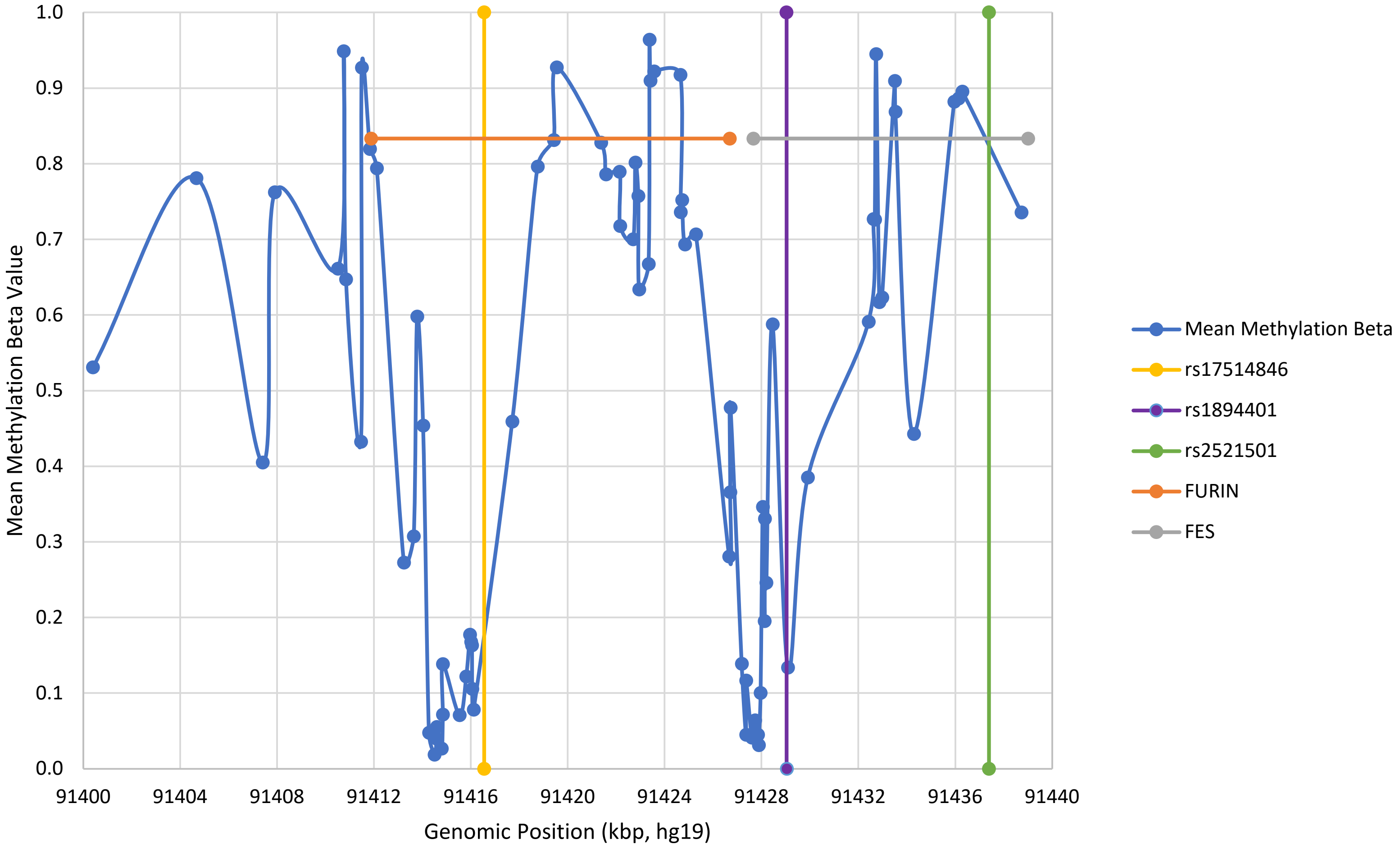

Supplementary Figure 3. Relative levels of DNA methylation at CpG sites in the genomic region containing the genetic variant rs1894401 and the *FES* gene

Each blue dot represents the mean beta value of a CpG site (calculated from 3 HUASMC and 1 HAoSMC cell lines). Vertical lines indicate the position of CAD-associated SNPs and horizontal lines indicate the position of the *FURIN* and *FES* genes.

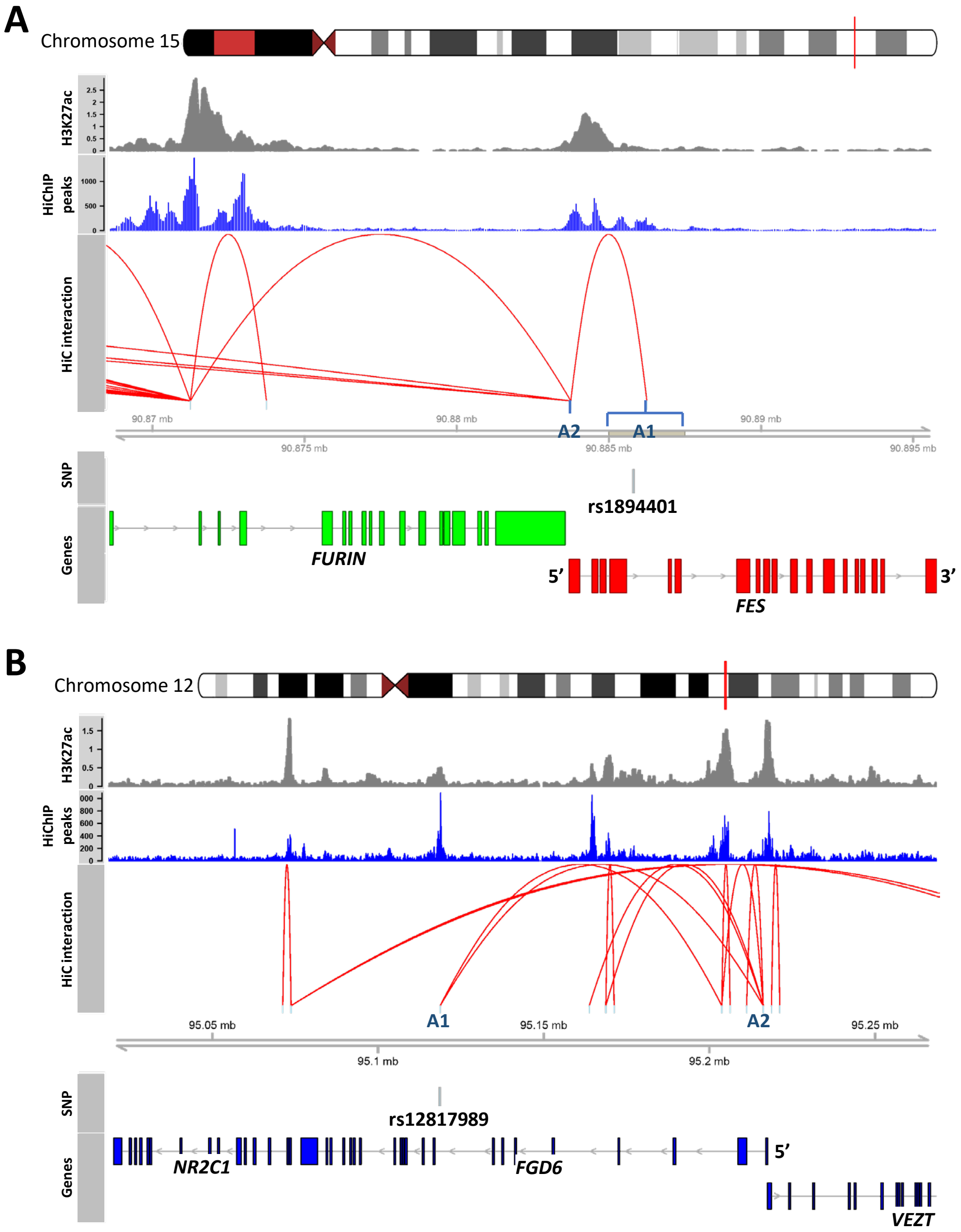

Supplementary Figure 4. Representative results from vascular smooth muscle cell H3K27ac HiChIP-seq analysis

A. Figure shows that the CAD-associated variant rs1894401 is in an anchor (A1) and the *FES* gene 5'-region is in a linked anchor (A2).

B. Figure shows that the CAD-associated variant rs12817989 is an anchor (A1) and the *FGD6* gene 5'-region in a linked anchor (A2).

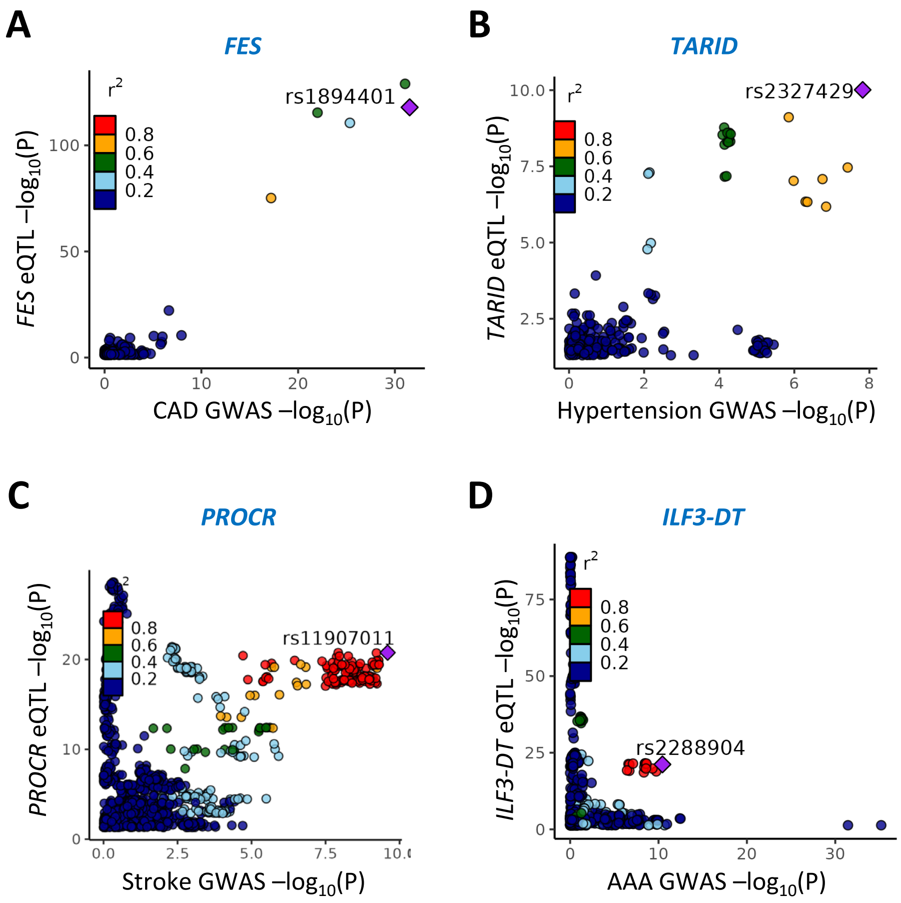

Supplementary Figure 5. Representative results of colocalization analyses

Scatter plots showing colocalization of GWAS signals with VSMC eQTL signals at selected representative loci associated with CAD (**A**), hypertension (**B**), stroke (**C**), or AAA (**D**).

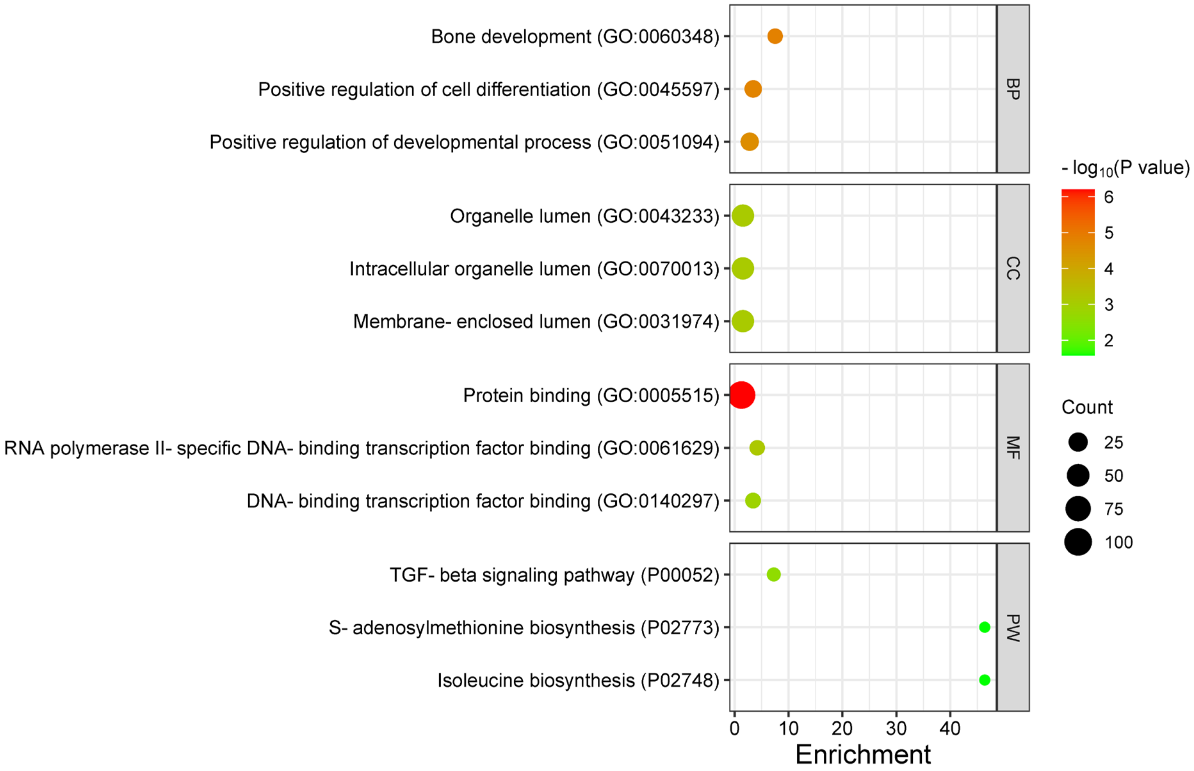

Supplementary Figure 6. Gene ontology enrichment of CAD candidate causal genes
BP: biological processes; CC: cellular component; MF: molecular function; PW: pathway.
TGF: transforming growth factor.

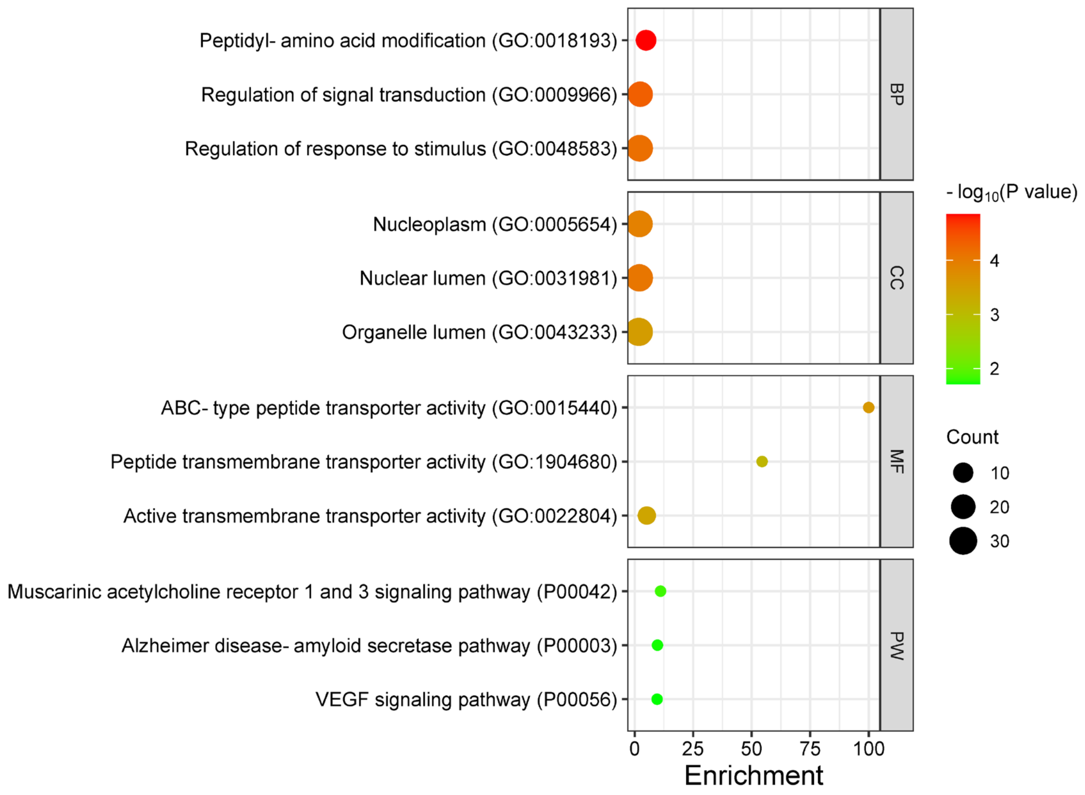

Supplementary Figure 7. Gene ontology enrichment of hypertension candidate causal genes
BP: biological processes; CC: cellular component; MF: molecular function; PW: pathway; VEGF: vascular endothelial growth factor.

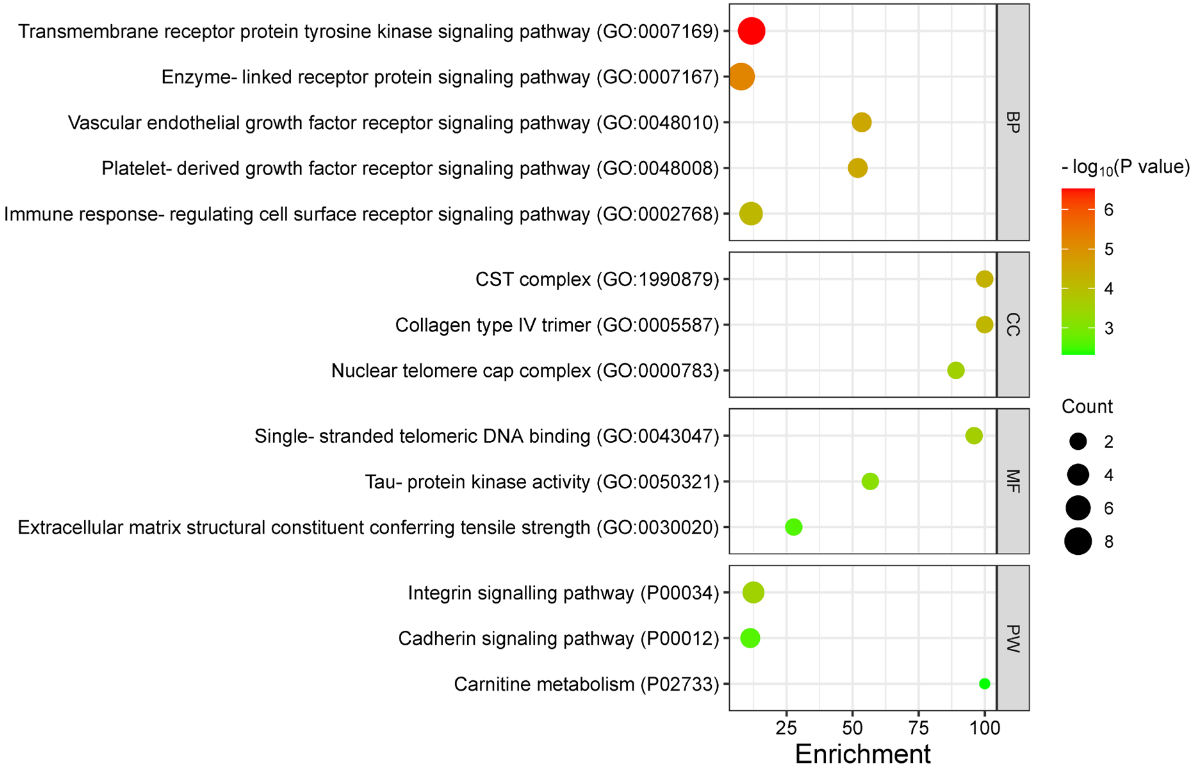

Supplementary Figure 8. Gene ontology enrichment of AAA candidate causal genes

BP: biological processes; CC: cellular component; MF: molecular function; PW: pathway.

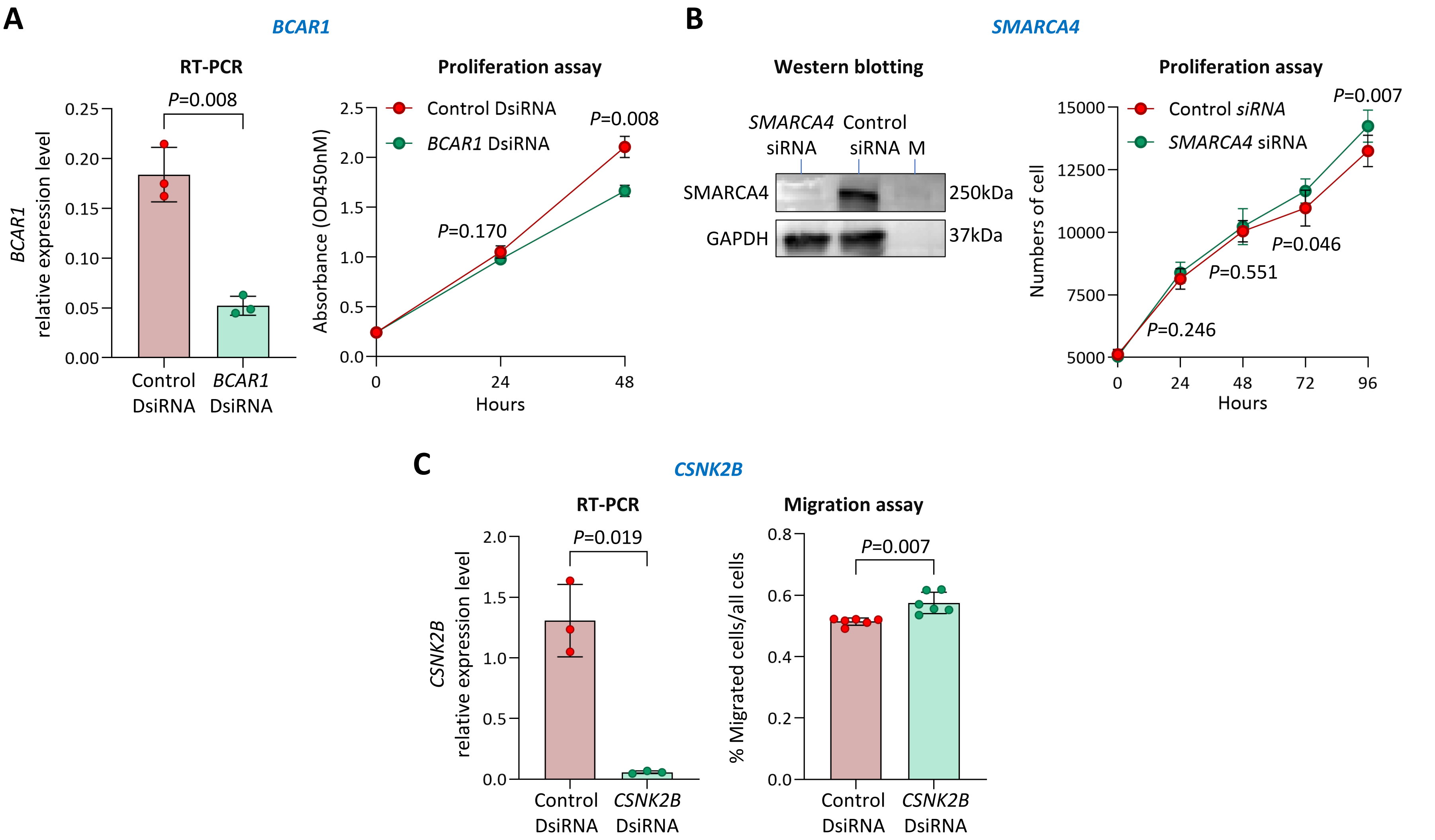

Supplementary Figure 9. *BCAR1* Knockdown decreases VSMC proliferation, *SMARCA4* Knockdown increases VSMC proliferation, whilst CSNK2B knockdown promotes VSMC migration

A. Results of *BCAR1* reverse transcriptase polymerase chain reaction (RT-PCR, left, n=3 technical replicates) and proliferation assay (right, n=3 technical replicates) of VSMCs transfected with either *BCAR1* DsiRNA or control DsiRNA. **B**. Results of Western blotting (left) and proliferation assay (right, n=8 technical replicates) of VSMCs transfected with either *SMARCA4* siRNA or control siRNA. Western blot image shown is a representative from 3 experiments. M, molecular weight marker. **C**. Results of *CSNK2B* RT-PCR (left, n=3 technical replicates) and migration assay (right, n=6 technical replicates) of VSMCs transfected with either *CSNK2B* DsiRNA or control DsiRNA. **A**-**C**. Charts show mean ± standard deviation (error bars); *P*-values are from two-sided t-test; source data are provided as a Source Data file.

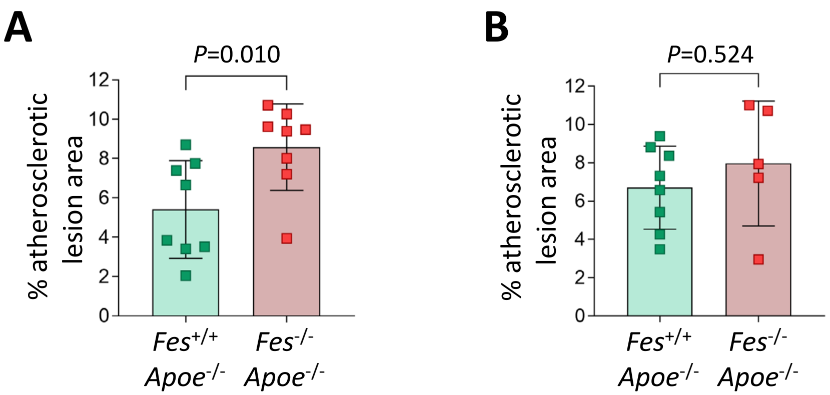

Supplementary Figure 10. ***Atherosclerotic lesions in male and female mice with and without Fes knockout***

A. Results of en face Oil-red-O staining of aortae of male mice. **B**. Results of en face Oil-red-O staining of aortae of female mice. Columns and error bars show mean ± standard deviation; *P*-values are from two-sided Mann-Whitney test; n=8 mice/group in A and n=5-8 mice/group in B.

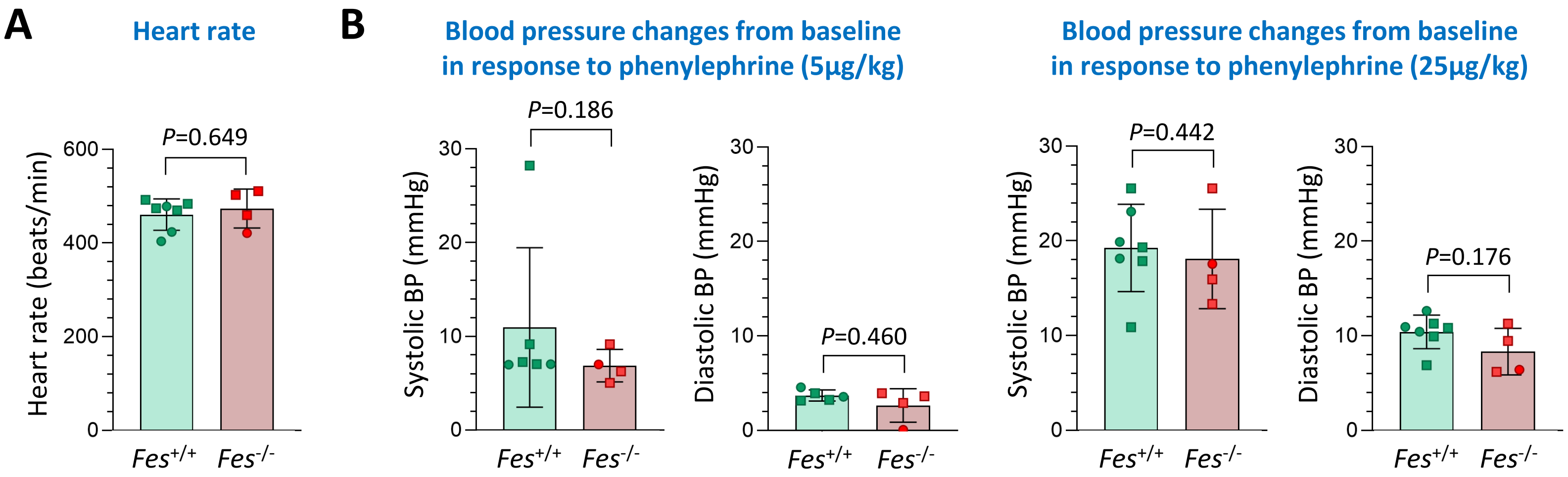

Supplementary Figure 11. Baseline heart rates, and blood pressure changes from baseline in response to phenylephrine treatment, of *Fes*$^{+/+}$ mice and *Fes*$^{-/-}$ mice

Square symbols represent values from male mice whilst circle symbols represent values from female mice; columns and error bars show mean ± standard deviation; *P*-values are from two-sided Mann-Whitney test; n=4-7 mice per group.

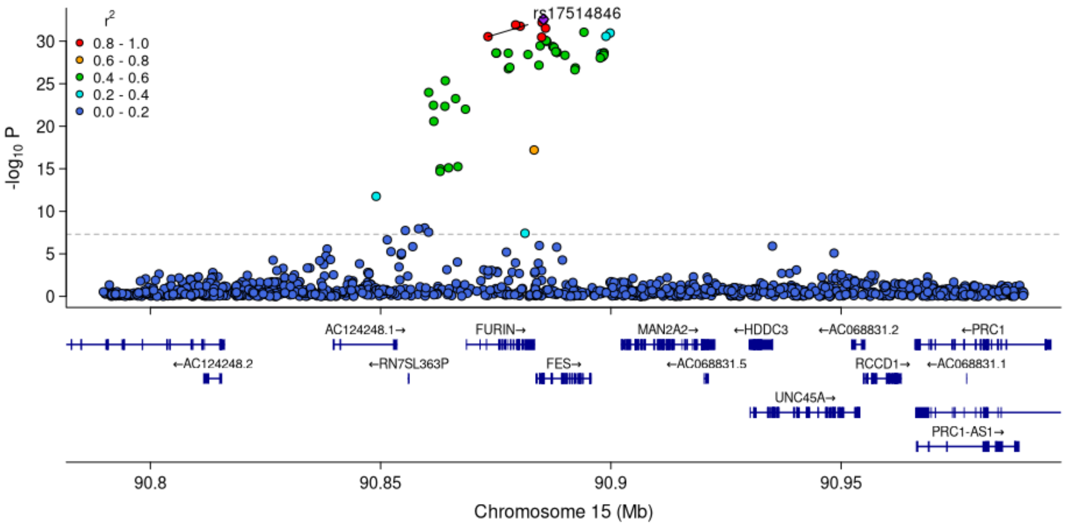

Supplementary Figure 12. Locus zoom plot of a coronary artery disease-associated locus on chromosome 15

The purple diamond symbol indicates the tagging single nucleotide polymorphism (SNP) rs17514846 that has been reported to be associated with coronary artery disease in genome-wide association studies (PMID: 29212778, 26343387). Each colored circle represents a SNP; the different colors indicate the different degrees of linkage disequilibrium (r^2) with the tagging SNP as shown in the legend . The Y-axis shows $–\log_{10}$ transformed P values for association with coronary artery disease.

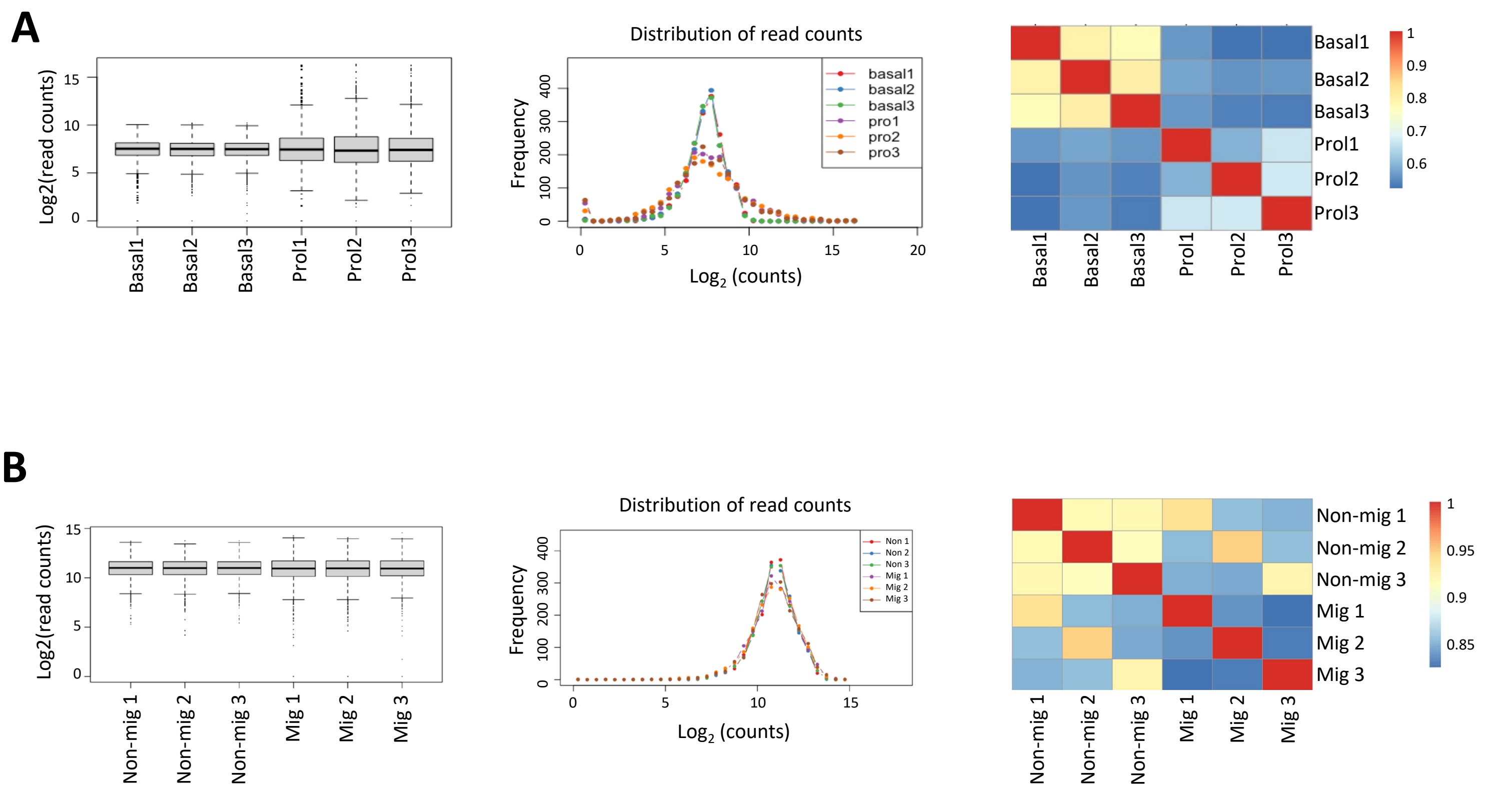

Supplementary Figure 13. Distributions and corrections of data from pooled CRISPR screen experiments

A. Data from vascular smooth muscle cell proliferation experiments. Left and middle panels: the baseline cell samples have similar distributions, as do the proliferated cell samples. Right panel: data of baseline cell samples have high corrections (indicating high reproductivity), as do data of proliferated cell samples. **B**. Data from vascular smooth muscle cell migration experiments. Left and middle panels: data of non-migrated (Non-mig) cell samples have similar distributions, as do data of migrated (Mig) cell samples. Right panel: data of non-migrated cell samples have high corrections (indicating high reproductivity), as do data of migrated cell samples.
