# Supplementary material for: Integrative functional genomics analysis identifies pleiotropic genes for vascular diseases
Source: Nat Commun. 2026 Feb 5;17:3376. doi: 10.1038/s41467-026-69273-8 (PMC13066215; doi:10.1038/s41467-026-69273-8)
Supplement: Supplementary file 2 — Description of Additional Supplementary Files [file 41467_2026_69273_MOESM2_ESM.pdf]

Description of Additional Supplementary Files

Supplementary Data 1. eQTLs of blood pressure/hypertension-associated genetic variants

P-values were derived from two-sided linear additive regression analysis with adjustment for sex, the first 2 principal components and the first 10 PEER (Probabilistic Estimation of Expression Residuals) factors as covariates.

Supplementary Data 2. eQTLs of stroke-associated genetic variants

P-values were derived from two-sided linear additive regression analysis with adjustment for sex, the first 2 principal components and the first 10 PEER (Probabilistic Estimation of Expression Residuals) factors as covariates.

Supplementary Data 3. eQTLs of abdominal aortic aneurysm-associated genetic variants

P-values were derived from two-sided linear additive regression analysis with adjustment for sex, the first 2 principal components and the first 10 PEER (Probabilistic Estimation of Expression Residuals) factors as covariates.

Supplementary Data 4. eQTLs of coronary artery disease-associated genetic variants

P-values were derived from two-sided linear additive regression analysis with adjustment for sex, the first 2 principal components and the first 10 PEER (Probabilistic Estimation of Expression Residuals) factors as covariates.

Supplementary Data 5. Colocalization of coronary artery disease GWAS signals with vascular smooth muscle cell eQTL signals

GWAS P-values were derived from two-sided logistic regression; eQTL P-values were from two-sided linear additive regression analysis with adjustment for sex, the first 2 principal components and the first 10 PEER (Probabilistic Estimation of Expression Residuals) factors as covariates; SMR P-values were from two-sided Wald test; HEIDI P-values were from two-sided heterogeneity test.

Supplementary Data 6. Colocalization of hypertension GWAS signals with vascular smooth muscle cell eQTL signals

GWAS P-values were derived from two-sided logistic regression (hypertension) or two-sided linear regression (BP); eQTL P-values were from two-sided linear additive regression analysis with adjustment for sex, the first 2 principal components and the first 10 PEER (Probabilistic Estimation of Expression Residuals) factors as covariates; SMR P-values were from two-sided Wald test; HEIDI P-values were from two-sided heterogeneity test.

Supplementary Data 7. Colocalization of stroke GWAS signals with vascular smooth muscle cell eQTL signals

GWAS P-values were derived from two-sided logistic regression; eQTL P-values were from two-sided linear additive regression analysis with adjustment for sex, the first 2 principal components and the first 10 PEER (Probabilistic Estimation of Expression Residuals) factors as covariates; SMR P-values were from two-sided Wald test; HEIDI P-values were from two-sided heterogeneity test.

Supplementary Data 8. Colocalization of abdominal aortic aneurysm GWAS signals with vascular smooth muscle cell eQTL signals

GWAS P-values were derived from two-sided logistic regression; eQTL P-values were from two-sided linear additive regression analysis with adjustment for sex, the first 2 principal components and the first 10 PEER (Probabilistic Estimation of Expression Residuals) factors as covariates; SMR P-values were from two-sided Wald test; HEIDI P-values were from two-sided heterogeneity test.

Supplementary Data 9. Vascular disease likely causal variants residing in open chromatin regions

Supplementary Data 10. Vascular disease likely causal variants and nearby CpG sites

Supplementary Data 11. Likely causative variants residing at H3K27ac HiChIP anchor and Likely causal genes situating at linked anchor

Supplementary Data 12. Likely causative variants residing in regions of open chromatin (ATAC-seq peak), H3K27ac and low DNA methylation

GWAS P-values were derived from two-sided logistic regression (CAD, hypertension, stroke, and AAA, respectively) or two-sided linear regression (BP); eQTL P-values were from two-sided linear additive regression analysis with adjustment for sex, the first 2 principal components and the first 10 PEER (Probabilistic Estimation of Expression Residuals) factors as covariates; SMR P-values were from two-sided Wald test; HEIDI P-values were from two-sided heterogeneity test.

Supplementary Data 13. Results of colocalisation analyses using coronary artery tissues or aortic artery tissues

PrediXcan P-values were from two-sided logistic regression (CAD, hypertension, stroke, and AAA, respectively) or two-sided linear regression (BP); SMR P-values were from two-sided Wald test; FUSION P-values were from two-sided Z-test.

Supplementary Data 14. Genetic correlations between coronary artery disease (CAD), blood pressure, stroke, and abdominal aortic aneurysm (AAA)

P-values were from two-sided Z-test.

Supplementary Data 15. Associations of candidate causal gene protein-coding variants with coronary artery disease and/or hypertension

Gene-phenotype association data from the UK Biobank and Finnish Biobank were obtained through searching the AstraZeneca PheWAS Portal (https://azphewas.com/) and PheWeb (https://r8.finngen.fi/). Data of functional consequence prediction (by SIFT, PolyPhen, CADD, REVEL, MetaLR, and Mutation Assessor) were taken from Ensembl Genome Brower 110 (www.ensembl.org). [1]Significance threshold: UK Biobank $P < 1\times10^{-8.7}$, Finnish Biobank P $<5^{\times10\text{-}8}$, from two-sided logistic regression analysis. [2]SIFT (sorts intolerant from tolerant substitutions), PMID: 11337480. [3]PolyPhen (polymorphism phenotyping), PMID: 12202775. [4]CADD (combined annotation dependent depletion), PMID: 24487276. [5]REVEL (rare exome variant ensemble learner), PMID: 27666373. [6]MetaLR, PMID: 25552646. [7]Mutation Assessor, PMID: 17976239.

Supplementary Data 16. Druggability of candidate causal genes for coronary artery disease

Supplementary Data 17. Drug-gene interaction of candidate causal genes for coronary artery disease

Supplementary Data 18. Druggability of candidate causal genes for hypertension

Supplementary Data 19. Drug-gene interaction of candidate causal genes for hypertension

Supplementary Data 20. Druggability of candidate causal genes for stroke

Supplementary Data 21. Drug-gene interaction of candidate causal genes for stroke

Supplementary Data 22. Druggability of candidate causal genes for abdominal aortic aneury

Supplementary Data 23. Drug-gene interaction of candidate causal genes for abdominal aortic aneurysm

Supplementary Data 24. Results of pooled CRISPR-Cas9-nuclease screen for genes affecting vascular smooth muscle cell proliferation

P-values from two-sided permutation test.

Supplementary Data 25. Results of pooled CRISPR-Cas9-nuclease screen for genes affecting vascular smooth muscle cell migration

P-values from two-sided permutation test.

Supplementary Data 26. Results of phosphoproteomics analysis of vascular smooth muscle cells transfected with control siRNA or FES siRNA

P-value from two-sided student's t-test.

Supplementary Data 27. RNA-sequencing results (transcript level) of vascular smooth muscle cells transfected with control siRNA or FES siRNA

Supplementary Data 28. RNA-sequencing results (gene level) of vascular smooth muscle cells transfected with control siRNA or FES siRNA

P-value from two-sided student's t-test.

Supplementary Data 29. Results of proteomics analysis of vascular smooth muscle cells transfected with control siRNA or FES siRNA

P-value from two-sided student's t-test.

Supplementary Data 30. Selection of blood pressure/hypertension-associated SNPs

Supplementary Data 31. Selection of stroke-associated SNPs

Supplementary Data 32. Selection of abdominal aortic aneurysm-associated SNPs

Supplementary Data 33. PCR primers used in the pooled CRISPR-Cas9-nuclease screen experiments

Supplementary Data 34. DsiRNA sequencecs

Supplementary Data 35. RT-PCR primers used in the FES knockdown experiments

Supplementary Data 36. Antibodies used in Western blot analysis
