# Supplementary material for: Integrative functional genomics analysis identifies pleiotropic genes for vascular diseases
Source: Nat Commun. 2026 Feb 5;17:3376. doi: 10.1038/s41467-026-69273-8 (PMC13066215; doi:10.1038/s41467-026-69273-8)
Supplement: Supplementary file 6 — Reporting Summary [file 41467_2026_69273_MOESM6_ESM.pdf]

nature portfolio

Corresponding author(s): Shu Ye

Last updated by author(s): Jan 26, 2026

Reporting Summary

Nature Portfolio wishes to improve the reproducibility of the work that we publish. This form provides structure for consistency and transparency in reporting. For further information on Nature Portfolio policies, see our Editorial Policies and the Editorial Policy Checklist.

Statistics

For all statistical analyses, confirm that the following items are present in the figure legend, table legend, main text, or Methods section.

n/a	Confirmed	
☐	☒	The exact sample size (*n*) for each experimental group/condition, given as a discrete number and unit of measurement
☐	☒	A statement on whether measurements were taken from distinct samples or whether the same sample was measured repeatedly
☐	☒	The statistical test(s) used AND whether they are one- or two-sided<br>*Only common tests should be described solely by name; describe more complex techniques in the Methods section.*
☒	☐	A description of all covariates tested
☐	☒	A description of any assumptions or corrections, such as tests of normality and adjustment for multiple comparisons
☐	☒	A full description of the statistical parameters including central tendency (e.g. means) or other basic estimates (e.g. regression coefficient) AND variation (e.g. standard deviation) or associated estimates of uncertainty (e.g. confidence intervals)
☐	☒	For null hypothesis testing, the test statistic (e.g. *F*, *t*, *r*) with confidence intervals, effect sizes, degrees of freedom and *P* value noted<br>*Give P values as exact values whenever suitable.*
☒	☐	For Bayesian analysis, information on the choice of priors and Markov chain Monte Carlo settings
☒	☐	For hierarchical and complex designs, identification of the appropriate level for tests and full reporting of outcomes
☒	☐	Estimates of effect sizes (e.g. Cohen's *d*, Pearson's *r*), indicating how they were calculated

Our web collection on statistics for biologists contains articles on many of the points above.

Software and code

Policy information about availability of computer code

Data collection	N/A
Data analysis	LDlink's SNPclip program (https://ldlink.nih.gov/), second-generation PLINK, LocusCompare V2 (https://www.locuscompare2.com/), Galaxy (https://usegalaxy.eu and https://usegalaxy.org), HiChIP pipeline by Dovertail Genomics (https://hichip.readthedocs.io/en/latest/index.html), Burrows-Wheeler Aligner (http://arxiv.org/abs/1303.3997), FitHiChIP4 (version 11.0), Givz (version 3.20), Gene Ontology (http://geneontology.org/), SRplot (https://www.bioinformatics.com.cn/srplot), LDSC (v1.0.1), AstraZeneca PheWAS Portal (https://azphewas.com/), PheWeb (https://r8.finngen.fi/), Ensembl Genome Browser 110 (www.ensembl.org), MAGeCK90 (version 0.5.9.5).

For manuscripts utilizing custom algorithms or software that are central to the research but not yet described in published literature, software must be made available to editors and reviewers. We strongly encourage code deposition in a community repository (e.g. GitHub). See the Nature Portfolio guidelines for submitting code & software for further information.

Data

Policy information about availability of data

All manuscripts must include a data availability statement. This statement should provide the following information, where applicable:

- Accession codes, unique identifiers, or web links for publicly available datasets
- A description of any restrictions on data availability
- For clinical datasets or third party data, please ensure that the statement adheres to our policy

The data that support the findings of this study are available from the corresponding author upon reasonable request. Source data are provided with this paper. The

RNA-Seq data and H3K27ac HiChIP-seq data are available from Gene Expression Omnibus with the accession number GSE189300 and GSE282557. The DNA methylation data are available from ArrayExpress with the accession ID E-MTAB-15426. The proteomics data and phosphoproteomics data are available from MassIVE with the accession IDs PXD061984 and PXD061992, respectively.

Research involving human participants, their data, or biological material

Policy information about studies with human participants or human data. See also policy information about sex, gender (identity/presentation), and sexual orientation and race, ethnicity and racism.

Reporting on sex and gender	The vascular smooth muscle cell bank used in this study includes arterial smooth muscle cells derived from umbilical cord tissues from male and female individuals. Information on sex was collected on hospital records. Sex was not considered in the study design. Results of eQTL analyses performed on the male and female samples separately are shown in Supplementary Table 1-4.
Reporting on race, ethnicity, or other socially relevant groupings	N/A
Population characteristics	N/A
Recruitment	N/A
Ethics oversight	The study was approved by the East Midlands - Derby Research Ethics Committee.

Note that full information on the approval of the study protocol must also be provided in the manuscript.

Field-specific reporting

Please select the one below that is the best fit for your research. If you are not sure, read the appropriate sections before making your selection.

[x] Life sciences [ ] Behavioural & social sciences [ ] Ecological, evolutionary & environmental sciences

For a reference copy of the document with all sections, see nature.com/documents/nr-reporting-summary-flat.pdf

Life sciences study design

All studies must disclose on these points even when the disclosure is negative.

Sample size	A previous publication (PMID: 23715323) by the GTEx Consortium indicated that a sample size of n>1000 will have >0.95 power for cis-eQTL analysis with alpha = 0.05/200,000, reflecting Bonferroni correction for 200,000 hypotheses based on 20,000 genes and an average of 10 non-redundant SNPs in a region ±100 kb of each gene. Our study was performed on a vascular smooth muscle cell bank that has a sample size of n>1000 (n=1,486).
Data exclusions	No data were excluded from the analyses.
Replication	The analyses were performed on a large collection of vascular smooth muscle cells from different individuals (n=1,486). We are unaware of another vascular smooth muscle cell collection of a similar large sample size, making replication unfeasible.
Randomization	Randomization was not relevant to this study as the samples were not allocated into groups.
Blinding	Blinding was not relevant to this study as the sample was not divided into groups.

Reporting for specific materials, systems and methods

We require information from authors about some types of materials, experimental systems and methods used in many studies. Here, indicate whether each material, system or method listed is relevant to your study. If you are not sure if a list item applies to your research, read the appropriate section before selecting a response.

Materials & experimental systems

n/a	Involved in the study	
[ ]	[x]	Antibodies
[ ]	[x]	Eukaryotic cell lines
[x]	[ ]	Palaeontology and archaeology
[ ]	[x]	Animals and other organisms
[x]	[ ]	Clinical data
[x]	[ ]	Dual use research of concern
[x]	[ ]	Plants

Methods

n/a	Involved in the study	
[ ]	[x]	ChIP-seq
[x]	[ ]	Flow cytometry
[x]	[ ]	MRI-based neuroimaging

Antibodies

Antibodies used	Rabbit snti-SMARCA4 (anti-BRG1), Abcam ab110641; 1:5000 dilution.<br>Mouse anti-FES, Proteintech 67524-1-IG; 1:1000 dilution.<br>Rabbit anti-MMP1, Abcam ab137332; 1:2000 dilution.<br>Rabbit anti-ACTA2, Proteintech 14395-1-AP; 1:2000 dilution.<br>Rabbit anti-SM22a (TAGLN), Proteintech 10493-1-AP; 1:2000 dilution.<br>Mouse anti-ACTB, Sigma a1978; 1:10000 dilution.<br>Mouse anti-VCL, Proteintech 66305-1-IG; 1:5000 dilution.<br>Rabbit anti-ALS2CR8 (CARF), Immunoway YT0206; 1:1000 dilution.<br>Rabbit anti-GAPDH, Cell Signaling Technology 2118; 1:5000 dilution.<br>Rabbit anti-GAPDH, Cell Signaling Technology 5174; 1:3000 dilution.<br>Rabbit anti-mouse IgG, HRP-linked, Sigma SAB3701083; 1:10000 dilution.<br>Goat anti-Rabbit IgG, HRP-linked, Abcam ab205718; 1:5000 dilution.<br>Goat anti-Rabbit IgG, HRP-linked, Boster Bio BA1054; 1:20000 and 1:40000 dilution.
Validation	Rabbit anti-SMARCA4, Abcam ab110641: the manufacturer's website indicates that specificity of this antibody has been confirmed by Western blot testing in SMARCA4 (BRG1) knockout HEK-293T cells.<br>Mouse anti-FES, Proteintech 67524-1-IG: the manufacturer's website indicates that this antibody is validated in WB, IF/ICC, ELISA and shows reactivity with human, mouse, rat samples.<br>Rabbit anti-MMP1, Abcam ab137332: the manufacturer's website indicates that this antibody is suitable for WB, ICC/IF and reacts with human and mouse samples.<br>Rabbit anti-ACTA2, Proteintech 14395-1-AP: the manufacturer's website indicates that this antibody is validated in WB, IHC, IF/ICC, IF-P and ELISA, and shows reactivity with human, mouse and rat samples.<br>Rabbit anti-SM22a (TAGLN), Proteintech 10493-1-AP: the manufacturer's website indicates that this antibody is suitable for WB, IHC, IF/ICC, IF-P, FC (Intra), IP, CoIP, ELISA and shows reactivity with human, mouse and rat samples.<br>Mouse anti-ACTB, Sigma a1978: the manufacturer's website indicates that this antibody is suitable for WB and ICC/IF, and shows reactivity with samples of human, mouse, rat and several other species.<br>Mouse anti-VCL, Proteintech 66305-1-IG: the manufacturer's website indicates that this antibody is validated in WB, IHC, IF/ICC, and FC, and shows reactivity with human, mouse, rat and pig samples.<br>Rabbit anti-ALS2CR8 (CARF), Immunoway YT0206: the manufacturer's website indicates that this antibody is suitable for WB, IHC, IF and flow cytometry, and reacts with human, mouse, rat, monkey, bovine, and pig samples.<br>Rabbit anti-GAPDH, Cell Signaling Technology 2118: the manufacturer's website indicates that this antibody is suitable for WB, IHC, and IF and shows reactivity with human, mouse, rat and monkey samples.<br>Rabbit anti-GAPDH, Cell Signaling Technology 5174: the manufacturer's website indicates that this antibody is suitable for WB, IHC, and IF and shows reactivity with human, mouse, rat and monkey samples.

Eukaryotic cell lines

Policy information about cell lines and Sex and Gender in Research

Cell line source(s)	Umbilical cord artery smooth muscle cells isolated from umbilical cord tissues from 1499 donors.<br>Human aortic smooth muscle cells (Lonza, CC-2571);<br>Human coronary artery SMCs (Cell Applications, 350K-05a).
Authentication	None of the cell lines used was authenticated.
Mycoplasma contamination	The cells were not tested for mycoplasma contamination
Commonly misidentified lines<br>(See ICLAC register)	N/A

Animals and other research organisms

Policy information about studies involving animals; ARRIVE guidelines recommended for reporting animal research, and Sex and Gender in Research

Laboratory animals	Fes−/− mice were backcrossed onto a C57BL6/J background for 7 generations before being intercrossed with Apoe−/− mice (B6.129P2-Apoetm1Unc/J) to generate Fes−/−/Apoe−/− mice. In the study of atherosclerosis, Fes-/-/Apoe-/- and littermate control (Fes+/+/Apoe-/-) mice of 6 weeks of age were fed a high fat diet for 12 weeks. In the blood pressure study, Fes-/-/Apoe-/- and littermate control (Fes+/+/Apoe-/-) mice of 8 weeks of age were subjected to blood pressure measurements.
Wild animals	N/A
Reporting on sex	The study included both male and female animals. The sex of each is indicated in the relevant figures. Sex was not considered in the study design. Sex-based analyses were not performed due to the relatively small sample sizes.
Field-collected samples	The study did not involve samples collected in the field.

Ethics oversight | The study was conducted in accordance with the UK Animals (Scientific Procedures) Act 1986, under a Home Office Project License (60/4332).

Note that full information on the approval of the study protocol must also be provided in the manuscript.

Plants

Seed stocks | N/A

Novel plant genotypes | N/A

Authentication | N/A

ChIP-seq

Data deposition

- [x] Confirm that both raw and final processed data have been deposited in a public database such as GEO.
- [x] Confirm that you have deposited or provided access to graph files (e.g. BED files) for the called peaks.

Data access links
May remain private before publication.
GSE282557

Files in database submission
GSE282557_FITHiChIP_SMC_5kb.interactions_FitHiC_Q0.01_MergeNearContacts_filteredwDistance.bed.gz (Bed) Interaction File;
GSE282557_SmoothMusHiChIP.hic (HiC) Juicer .hic File.

Genome browser session
(e.g. UCSC)
https://tiny.one/3tmm2cnd

Methodology

Replicates
Two independent H3K27ac ChIP-Seq on primary human coronary artery smooth muscle cells were performed, prior to enriching ligated junctions as per Hi-C Protocol Library preparation.

Sequencing depth
>300 million paired-end, 2X151bp reads

Antibodies
Anti-Histone H3 (acetyl K27) antibody (Abcam, ab4729)

Peak calling parameters
Raw Fastq format paired-end reads were processed with HiChIP pipeline by Dovertail Genomics (https://hichip.readthedocs.io/en/latest/index.html). The hg38 human genome index generated with Burrows-Wheeler Aligner (http://arxiv.org/abs/1303.3997) was used as reference for alignments and valid pair selection. Duplicates pairs were removed using pairtools.

The FitHiChIP execution was configured to analyze peak-to-all interactions (interaction type 3). Bin sizes were ran in 2.5Kb, 5Kb, 10Kb, 15Kb, and 20Kb (Size of the bins=2500,5000,10,000,15000,20000), respectively. The FitHiChIP execution was configured to analyze peak-to-all interactions (interaction type 3). Bin sizes were ran in 2.5Kb, 5Kb, 10Kb, 15Kb, and 20Kb (Size of the bins=2500,5000,10,000,15000,20000). The analysis ran with equal occupancy binning with a lower distance threshold of 1,000 bp and an upper distance threshold of 80,000,000 bp for calling significant interactions with false discovery rate (FDR) threshold of 0.01 (QVALUE=0.01). 200 bins were used for spline modeling in FitHiChIP. Bias correction was enabled (BiasType=1), utilizing a coverage-specific bias correction method (1:coverage specific). A regression model was applied to adjust for bias based on observed contact counts and bias values. Additionally, nearby interactions were merged (MergeInt=1) to refine the interaction landscape. The loop calling peak file was SRX7805852 peak call(q<1E-05) from GSM4340246 in which contains 32107 peaks.

Data quality
The HiChIP peak data quality assessment indicates a total of 57,887 ChIP peaks, with an average peak size of 1,036 bp and a median peak size of 630 bp. The enrichment of reads around peak centers is robust, with 26,339,766 reads (4.86%) mapped within a 500 bp window, 38,894,308 reads (7.18%) within 1,000 bp, and 53,801,571 reads (9.94%) within 2,000 bp around peak summits. The observed-to-expected ratio for read enrichment suggests strong signal specificity, with values of 5.39 for 500 bp, 3.98 for 1,000 bp, and 2.75 for 2,000 bp windows.

The HiChIP loop calling data quality was assessed at FDR 0.01 after merge. The 2.5kb analysis identified a total of 80,028 loops. The median loop distance was 17,500 bp, and the mean loop distance was 100,345.50 bp.The 5kb analysis identified a total of 81,108 loops. The median loop distance was 95,000 bp, and the mean loop distance was 174,608.30 bp. The 10kb analysis identified a total of 82,462 loops. The median loop distance was 170,000 bp, and the mean loop distance was 277,725.98 bp. The 15kb analysis identified a total of 78,061 loops. The median loop distance was 225,000 bp, and the mean loop distance was 375,683.12 bp.20 kb analysis identified a total of 72,009 loops. The median loop distance was 280,000 bp, and the mean loop distance was 483,911.46 bp.

Software

The hg38 human genome index was generated with Burrows-Wheeler Aligner (bwa). Raw HiChIP data was then using pairtools to record valid ligation events,sorting, removing PCR duplicates and split the final file to .pairs and bam files. The pairs file was used to generating contact matrix and the bam file was sorted and indexed using samtools. FitHiChIP (version 11.0) was used to call significant HiChIP loops at 2.5Kb, 5Kb, 10Kb, 15Kb, and 20Kb resolution.
